# Role of miRNA in the Regulatory Mechanisms of Estrogens in Cardiovascular Ageing

**DOI:** 10.1155/2018/6082387

**Published:** 2018-12-20

**Authors:** Daniel Pérez-Cremades, Ana Mompeón, Xavier Vidal-Gómez, Carlos Hermenegildo, Susana Novella

**Affiliations:** Department of Physiology, Faculty of Medicine and Dentistry, University of Valencia and INCLIVA Biomedical Research Institute, Valencia, Spain

## Abstract

Cardiovascular diseases are a worldwide health problem and are the leading cause of mortality in developed countries. Together with experimental data, the lower incidence of cardiovascular diseases in women than in men of reproductive age points to the influence of sex hormones at the cardiovascular level and suggests that estrogens play a protective role against cardiovascular disease and that this role is also modified by ageing. Estrogens affect cardiovascular function via their specific estrogen receptors to trigger gene expression changes at the transcriptional level. In addition, emerging studies have proposed a role for microRNAs in the vascular effects mediated by estrogens. miRNAs regulate gene expression by repressing translational processes and have been estimated to be involved in the regulation of approximately 30% of all protein-coding genes in mammals. In this review, we highlight the current knowledge of the role of estrogen-sensitive miRNAs, and their influence in regulating vascular ageing.

## 1. Introduction

The prevalence, incidence, and prognosis of cardiovascular diseases differ between genders. Indeed, statistical data reveal that women develop cardiovascular disease later than men [1] and the incidence of cardiovascular diseases in women increases from menopause [2, 3]. Both clinical and experimental data have extensively demonstrated the beneficial effects of estrogens at the cardiovascular level [4, 5], although hormonal replacement therapies (HRTs) in postmenopausal women have been implemented with controversial results [6, 7]. Such studies have led some researchers to conclude that the protective effect of HRT depends on age [8].

Ageing is a physiological and multifactorial process characterized by the progressive loss of anatomical and functional integrity, which leads to an increased risk of different pathologies, including cardiovascular disease. At the molecular level, different mechanisms have been established as crucial in the regulation of the ageing process [9]. Among these, epigenetic mechanisms affect gene expression without causing changes in the DNA sequence and can be influenced by external factors, including the environment and lifestyle [10].

Some evidence indicates that estrogen-dependent regulation of cardiovascular function in ageing is mediated by epigenetic mechanisms. An age-related increase in methylation-associated inactivation of genes encoding estrogen receptors (ERs) has been described, and ER methylation in atherosclerotic plaques is higher than in nonplaque regions in vascular tissues [11, 12], which suggests that estrogen activity in atherogenesis and vascular system aging is epigenetically regulated. Another epigenetic mechanism that has also been associated with estrogens is histone modification. In this regard, in a postmenopausal metabolic syndrome murine model, estradiol prevented cardiovascular dysfunction by suppressing histone H3 acetylation [13].

In addition to DNA methylation and histone modification, the most recently described epigenetic mechanism is RNA-based machinery. Regulatory noncoding RNAs are classified depending on RNA length, and among them, microRNAs (miRNAs) constitute the dominant class in most tissues. MiRNAs regulate protein translation by targeting their target messenger RNAs (mRNAs) via sequence-specific interaction to repress translation or degrade target mRNA [14]. In addition, emerging evidence suggests that miRNAs have also nuclear functions in the regulation of gene expression at transcriptional level [15]. Most miRNAs are located within cells, although some have also been found circulating in body fluids [16]. Thus, miRNA-mediated regulation is now considered one of the most important posttranscriptional gene regulation mechanisms and is estimated to modulate up to 30% of mammal genes, including important roles in human physiology, ageing, and cardiovascular function [17, 18].

Given the world's increasingly ageing population [19] and that cardiovascular diseases are the leading cause of death in developed countries, it is important to improve our understanding of the regulatory mechanisms underlying the ageing of the cardiovascular system. In addition, although growing evidence has established miRNAs as crucial epigenetic regulators of vascular function, their role in the regulation of estrogen in cardiovascular ageing has not yet been fully elucidated. Therefore, in this review, we summarize current knowledge of the role of estrogens in cardiovascular function and ageing with a special focus on the miRNAs related to estrogens during female vascular ageing.

## 2. The Involvement of Estrogens in Vascular Function

Estrogens modulate the cardiovascular system directly by acting on vascular and inflammatory cells, which express ERs, or indirectly via systemic effects [20]; estrogen functions through ERs by genomic and nongenomic mechanisms. In the former, also known as the “classical” mechanism, estrogens bind to ERs to form a complex that regulates gene transcription by binding to specific DNA motifs in gene promoter regions [21]. In this sense, ER*α* and ER*β* are the two main ER isoforms, and these form homo- or heterodimers, which can induce changes in gene expression. The involvement of specific ER isoforms in estrogen-mediated effects has been extensively studied [22], and both opposing gene expression regulatory effects [23, 24] and redundant mediatory roles [25, 26] have been described. Estrogen signaling is selectively regulated by the relative balance between ER*α* and ER*β* expression in target organs [27], although the beneficial effects that estrogens have on the vascular system are mainly attributed to ER*α* [28, 29]. Furthermore, estrogens can also trigger fast responses through plasma membrane ER receptors and G protein-coupled ERs (GPERs) [30].

In general, the vascular protective effects of estrogens are attributed to their role in increasing arterial vasodilation, their action on vasoactive mediator release and smooth muscle contraction, inhibition of inflammatory processes, and regulation of systemic lipid metabolism and oxidative stress balance [2, 31, 32]. The regulation of vascular reactivity by estrogens is mainly related to the maintenance of normal endothelial function [33]. In endothelial cells, estrogens modulate nitric oxide (NO) bioavailability by both genomic and nongenomic effects by increasing endothelial NO synthase (eNOS) expression at the transcriptional level, eNOS activation through phosphorylation, and regulation of its endogenous inhibitors and cellular location [34–36]. Moreover, a role for estrogens in regulating other vascular mediators related to prostanoids and endothelin signaling has also been described. Estradiol increases prostacyclin release by upregulating cyclooxygenase 1 (COX-1) and prostacyclin synthase (PGIS) expression in endothelial cells [37] and by decreasing endothelin-1 release in both basal and stimulated conditions [38, 39].

In addition to the regulation of endothelial-derived factors, estrogens directly regulate the smooth muscle layer by inhibiting the proliferation, migration, and vascular contraction of vascular smooth muscle cells (VSMCs) [40]. Indeed, estrogen-mediated vasorelaxation can also occur in endothelium-denuded segments [41]. Specifically, estradiol decreases smooth muscle constriction by interfering in the mechanisms of Ca^2+^ mobilization and Ca^2+^ entry [42] and by activating K^+^ channels [43], leading to membrane hyperpolarization and vascular relaxation. Estrogen can also modulate vasoconstriction by interfering in protein kinase C and Rho-kinase signaling in VSMCs [44, 45].

The renin-angiotensin system (RAS) is another important regulator of vascular contractibility which is regulated by estrogen. Estrogens are implicated in the inhibition of circulating renin, the activity of angiotensin-converting enzymes, and in circulating angiotensin (Ang) II levels [46]. Furthermore, components of RAS are synthesized and act locally in different tissues, including in the vasculature. In this case, estradiol increases the expression of Ang 1–7 by inducing the expression of angiotensin-converting enzymes [47] and of Ang II receptor type 1 expression [48] in endothelial cells, thus promoting vasodilation. In addition, estradiol-dependent NO production is mediated by Ang 1–7-induced activation of the Mas receptor [49], suggesting the presence of a functional interaction between both these pathways.

The beneficial effects of estrogens in the cardiovascular system are also attributed to their role in modulating the inflammatory response [50] and vascular lipid accumulation [32]. Estrogens inhibit monocyte-endothelial interactions by reducing the expression of cell adhesion molecules in the endothelium when exposed to inflammatory stimuli [51, 52]. Moreover, a reduction in neutrophil chemotaxis [53] and leukocyte infiltration [54] have been established as inflammatory regulatory mechanisms which are mediated by estrogens after vascular injury. Estradiol also reduces the expression of superoxide-induced adhesion molecules and cytokines in VSMCs by inhibiting nicotinamide adenine dinucleotide phosphate (NADPH) oxidase expression [55]. Moreover, it regulates oxidative stress by decreasing both the expression and activity of superoxide dismutase in VSMCs [56] and endothelial cells [57]. Estrogens can also confer protective effects by modulating systemic lipid metabolism [32], lipid-vascular wall interactions by reducing lipid loading [58, 59], and oxidative stress-mediated LDL modifications [60], thus preventing the formation of foam cells.

## 3. The Role of Estrogens in Vascular Ageing

Epidemiological data reveal sex differences in the number of deaths caused by cardiovascular disease. These numbers are greater in men than in women under the age of 65 and similar over this age [61], suggesting the importance of ageing in sex-related differences observed in cardiovascular disease. In this regard, sex-specific differences of cardiovascular ageing have been reported, and these patterns have been explained by both hormonal and nonhormonal factors [62].

Considering the beneficial role of estrogens in the cardiovascular systems described above, the use of HRT in postmenopausal women has produced controversial results [6, 7]. The current consensus indicates that the protective effects that estrogens confer on cardiovascular function depend on the prompt initiation of estrogen therapy after menopause [63]. The phenomenon is referred to as the “timing hypothesis” and postulates that estrogen supplementation may only have beneficial effects when initiated before the detrimental effects that ageing has on the cardiovascular system become established [8]. Vascular ageing is associated with endothelial dysfunction and arterial stiffening, vascular remodeling, and increased inflammation [64]. These characteristics can lead to pathological conditions such as myocardial hypertrophy, fibrotic tissue formation, and increased systolic pressure, resulting in a higher risk of atherosclerosis, hypertension, and ischemic cardiovascular disease [65]. Endothelial dysfunction is less prominent in premenopausal women compared to age-matched men and postmenopausal women, which highlights the protective effect of estrogens [66]. Indeed, in a recent study, Nicholson et al. demonstrated that the contractility responses of uterine arteries to estradiol treatment in postmenopausal women were impaired compared to their premenopausal counterparts [67].

The vascular ageing process is associated with a decline in endothelial-dependent relaxation and an increased vasoconstrictor responses mediated by an imbalance between vasodilator and vasoconstriction factors [68–70]. In this regard, NO and prostanoids seem to be the main pathways implicated, and these act by progressively reducing the bioavailability of NO and increasing vasoconstrictor prostanoids in aged vascular tissues. Indeed, relaxation induced by bradykinin in isolated mesenteric microvessels was mainly mediated by the action of NO, although COX-dependent vasodilators were also implicated in young subjects, whereas COX-dependent activity produced vasoconstriction in older subjects [69]. Although some of the data regarding eNOS expression at the molecular level are contradictory, ageing progressively decreases eNOS activity both in animal models and in human samples [71, 72]. Moreover, decreased expression of the eNOS cofactor, tetrahydrobiopterin [73], elevated circulating levels of endogenous inhibitors [74], and enhanced oxidative stress [69] have also been postulated as mechanisms which underlie decreased eNOS activity during ageing. Conversely, rather than altered expression of the COX isoforms, an age-associated switch from vasodilatory to vasoconstrictor prostanoid release has been related to enhanced expression of the thromboxane A2 (TXA2) receptor in the smooth muscle layer in both human mesenteric arteries and in aortic segments from female mice [69, 70, 75].

Oxidative stress is also increased in aged tissues, including the vascular system, and influences endothelial dysfunction by scavenging NO, thus decreasing its bioavailability [76]. Increased oxidative stress levels in vascular ageing are caused by altered antioxidant enzyme expression and enhanced production of reactive oxygen species (ROS) mainly by mitochondria and NADPH oxidase but also by COX and uncoupled eNOS [77]. The role of estrogens in oxidative stress during ageing has been previously reported: in vascular tissues, a decline in NO-mediated vasodilation is associated with increased ROS, especially superoxide, in coronary arterioles [78]. In the same study, the authors describe a decrease in Cu/Zn superoxide dismutase expression in both aged and ovariectomized rats, while estrogen replacement restores its expression to the levels of young intact animals [78]. Furthermore, COX-dependent superoxide production induced by a TXA2 analogue in female aortic segments was enhanced by ageing and estrogen deprivation, findings that were abrogated by estrogen supplementation [79].

Ageing also influences the structural properties of vasculature, and changes in the media thickness and extracellular fiber content contribute to low arterial compliance. Specifically, ageing is associated with the increased production of collagen fibers and decreased elastin fiber synthesis, vascular calcification, and VSMC proliferation [80]. Estrogen administration mitigates progressive age-dependent reductions both in human carotid artery wall thickness in postmenopausal women [81] and in the mesenteric arteries of old female rats [82]. In addition, increased collagen content has been associated with decreased activity and protein levels of specific collagen-degrading metalloproteinase enzymes [82, 83]. Finally, aberrant VSMC growth is also associated with ageing-associated remodeling processes as VSMCs switch from a contractile to synthetic-proliferative phenotype [84]. As previously described, estrogens inhibit the proliferation of cultured VSMCs [40] and it is thought that ageing males are more prone than females to these changes in the VSMC phenotype [85]. In addition, estrogen treatment also attenuates neointimal formation after vascular damage [86]. Finally, in relation to these metalloproteinase and VSMC phenotypes, estrogens may be involved in plaque instability and thus the greater cardiovascular disease risk in postmenopausal women who start HRT late.

Along with endothelial dysfunction and vascular remodeling, the other key player in vascular ageing is inflammation. Ageing is associated to a progressive increment of the tissue's proinflammatory status, phenomenon termed as “inflammageing.” The signaling of this low-grade chronic inflammation state is linked to an increased cell death, a metabolic dysfunction and a proteostasis loss, and it is also associated with an age-related functional detriment [87]. At vascular level, the ageing process is characterized by an acquisition of a proinflammatory phenotype with increased release of cytokines and enhanced cell adhesion molecules expression, developing a more adhesive endothelium, phenomenon also known as endothelial activation [64, 88]. The role of estrogens as an anti- and/or proinflammatory factor in this process is controversial and may be dependent on immune stimuli, the cell types involved in the response, organ target, timing and concentration of their administration, intracellular metabolism of estrogen, and relative expression of the implicated ERs [50]. In a study using uterine arteries from postmenopausal women that were exposed to estradiol, Novella et al. demonstrated that although estradiol exposure decreased most of the inflammatory cytokines assessed in women in the early stages of menopause, ageing was associated with estrogen's effect on the switch from anti-inflammatory to proinflammatory in the arteries of women who had started menopause at least 10 years prior [89], suggesting that the effect of estrogens is timing-dependent. In addition, estradiol attenuates inflammatory responses in macrophages and VSMCs derived from young but not old female mice [90], suggesting that the anti-inflammatory properties of estradiol are lost as ageing continues.

As mentioned above, the relative expression of specific ERs has been related to the role of estrogens in vascular ageing and it has postulated that a change in the balance between ER*α* and ER*β* could explain the lack of vascular protection provided by estrogen. In that sense, an age-dependent increase in ER*β* but not in ER*α* expression was observed in uterine arteries from postmenopausal women [89], thus producing this increased ER*β*/ER*α* ratio. In young ovariectomized mice, although estrogen supplementation downregulated ERs, it did not alter the ER*β*/ER*α* ratio in endothelium or smooth muscle; in contrast, the ER*β*/ER*α* ratio increased after estrogen treatment in aged female mice [91]. Finally, decreased ER*α* expression was also reported in aged compared to young macrophages [90].

## 4. Sex and Age Influence in miRNA Expression

Sex and age differences in miRNA expression profiles have been described in different tissues [92–94], providing evidence for the role of sex hormones and ageing in miRNA regulation. Sex differences are usually attributed to the modulation of estrogen transcriptional activity mediated by ERs, and so differences in the miRNA profiles obtained from ER+ and ER− breast cancer cells [95, 96] are related to the loss of ER binding sites located near the miRNA sequences which are found less abundance in ER− breast cancer cells [96]. At the vascular cell level, ER binding sites were located within the regulatory region of estradiol-regulated miRNAs [97, 98].

Sex-biased miRNAs are also driven by the expression of miRNAs located in sex chromosomes. It is worth noting that, according to miRBase (March 2018; http://www.mirbase.org), 118 miRNAs are located on the human X chromosome but only 4 were present on the Y chromosome. Most X chromosome miRNAs escape X chromosome inactivation which occurs early in development to compensate for gene dosage imbalances between the sexes [99]. In this respect, the mosaicism resulting from X chromosome inactivation may be involved in some of the increased susceptibility to inflammatory and autoimmune diseases experienced by women [100]. Regarding X-linked miRNAs and cardiovascular diseases, Florijn et al. recently reviewed the implication of miRNAs in women with heart failure with a preserved ejection fraction and concluded that estradiol-induced miRNAs are protective while X-linked miRNAs are associated with deleterious effects [101]. Furthermore, both age and estrogens can modulate miRNA profiles by regulating miRNA biosynthesis pathways.

miRNA production is a two-step process involving nuclear cropping and cytosolic dicing; mature miRNAs are derived from a stem-loop transcript (also known as pri-miRNA) which is cleaved in the nucleus by a microprocessor complex—comprising the RNase III, Drosha, and DiGeorge syndrome critical region 8 (DGCR8)—into a small hairpin-shaped RNA (pre-miRNA) which is transported to the cytoplasm through exportin 5 where maturation can be completed. In a second processing step, the pre-miRNA is cleaved—by the RNase III, Dicer—into approximately 22-nt miRNA duplexes. One strand from the miRNA duplex usually remains as a mature miRNA, while the other is generally selectively degraded via a thermodynamic stability-dependent process. Finally, mature miRNAs are loaded into Argonaute (AGO) proteins to form, along with other components, the RNA-induced silencing complex (RISC) effector. The final miRNAs function as a guide by base pairing—usually at the 3′-untranslated region (UTR)—to target mRNAs, and AGO proteins recruit factors that induce the translational repression of these mRNAs [102].

With ageing, molecules implicated in miRNA expression machinery become downregulated, thus suggesting that miRNA expression is age-dependent: an effect described in old versus young peripheral blood mononuclear cells [103]. The same study showed that the expression of miRNA biogenesis molecules is depressed in octogenarians compared with centenarians, which may indicate that greater expression of miRNA production components is associated with extraordinarily successful ageing. At the vascular level, Dicer1 is downregulated in old versus young cerebral vessels isolated from rats, which these authors associated with altered miRNA expression profiles and impaired endothelial function [104]. Indeed, the impaired vascular formation observed in Dicer1 knockout mice was one of the first pieces of evidence that related miRNA biosynthesis to vascular function [105]. In endothelial cells, Dicer1 depletion revealed that miRNA processing is essential for correct endothelial gene expression and function, including proliferation and angiogenesis [106, 107].

The role of estrogen in regulating key miRNA production molecules has been reviewed elsewhere [108], and of note, most studies relating estrogen activity and miRNA biosynthesis have been performed in reproductive organs. Differences in key miRNA-processing genes have been observed between ER+ and ER− breast cancer cells [95, 109]; specifically, the expression of Dicer1, DGCR8, and Drosha was higher, and that of Ago-2 was lower in ER+ breast tumors [110]. Nevertheless, among the miRNA processing genes, only Dicer1 contains an ER*α* binding site in its regulatory region [110]. Furthermore, a regulatory ER-Drosha interaction has been reported in breast cancer cells [111] and an estrogen-dependent increase in exportin-5 expression was reported in the mouse uteri [112]. At the vascular level, global transcriptomic analysis data of endothelial cells treated with estradiol reported the deregulation of key miRNA biosynthesis pathway genes [113]. Specifically, DGCR8 upregulation and Dicer1 and Ago-2 downregulation were observed in estradiol-treated cells, suggesting that estrogens regulate endothelial miRNA production machinery [113].

## 5. Estrogen-Regulated miRNAs and Vascular Ageing

As described above, sex- and age-specific miRNA profiles are the result of transcriptomic changes, sex chromosome expression, and miRNA biosynthesis regulation. In addition, findings in women receiving HRT have provided insights into the roles of estrogen-associated miRNAs during ageing in different tissues (Table 1). Changes in the miRNA expression profile in bone tissue from both ovariectomy-induced osteoporotic mice and postmenopausal women have been described [114, 115]. Specifically, miR-127 and miR-136 have been described as negative regulators of bone mass [114], whereas the expression level of miR-30b-5p has been proposed as a suitable serum biomarker for osteoporosis and osteopenia in postmenopausal women [115]. Furthermore, Olivieri et al. reported that estrogen has a positive impact in postmenopausal women using HRT as a result of skeletal muscle changes mediated via the suppression of miR-182 and miR-223 expression. These miRNAs are implicated in regulating the insulin/insulin-like growth factor (IGF-1) pathway which is key in muscle mass homeostasis [116]. Moreover, changes in the miRNA expression profile in adipose tissue have recently been described in association with HRT [117]. Specifically, miR-19a-3p was identified as being HRT-sensitive in adipose tissue because its levels in HRT-treated postmenopausal women were similar to those observed in premenopausal women and were higher than in postmenopausal women who did not use HRT. ESR1 is a miR-19a-3p target [118] and, thus, could be involved in the changes in ER*α* observed in adipose tissue during ageing [119] and may underlie the adverse age-related alterations in adipose metabolism in women [120].

In addition to tissue-specific miRNA expression, estrogen-dependent regulation in circulating miRNAs has also been described both in ovariectomized animals [115] and postmenopausal women receiving HRT [121, 122]. The studies in women were performed in monozygotic twin pairs and showed an association between changes in serum inflammatory markers and inflammatory-related miR-21 and miR-146a [121]. There were also alterations in the miRNAs included in exosomes in postmenopausal women using HRT [122], suggesting that changes in circulating miRNAs are associated with estradiol levels. Therefore, estrogen-sensitive miRNAs could be used both as therapeutic targets and as potential biomarkers for characteristic physiological alterations related to female ageing, such as osteoporosis, sarcopenia, changes in body fat homeostasis, and ageing-associated inflammation.

Specific miRNAs implicated in cardiac and vascular ageing have been reviewed in depth elsewhere [17, 18]. In addition, we have addressed the role of estrogen-regulated miRNAs in cardiovascular function in a recent review [113]. However, how miRNAs are implicated in the action of estrogen in the vasculature during ageing remains to be elucidated. In the following paragraphs, we describe the involvement of specific miRNAs in the effect of estrogens during female vascular ageing, and Tables 2 and 3 summarize the main circulating and tissue-specific miRNAs involved in female vascular ageing, respectively.

*miR-126* is highly expressed in vascularized tissues and was the first miRNA reported as being crucial in endothelial biology. It is located within an intron of the epidermal growth factor-like domain multiple 7 *(EGFL7)* gene which is mostly expressed in endothelium and is involved in vascular angiogenesis. This miR-126 is involved in vascular integrity and angiogenesis [123, 124] but also appears to modulate immune cell adhesion and VSMC function [125, 126]. Serum levels of estradiol are positively associated with miR-126-3p expression throughout the menstrual cycle; estradiol also increases miR-126-3p expression in cultured endothelial cells [127] but decreases it in lymphocytes [128], suggesting that it has a cell-specific effect on miR-126-3p expression. In endothelial cells, estradiol-dependent miR-126-3p suppresses the miR-126-3p targets, Spred1 and *VCAM1*, and is related to an increase in cell migration, proliferation, and tube formation, while decreases monocyte adhesion [127]. Moreover, miR-126-3p is also implicated in the estradiol-dependent reduction of plaque size in ApoE^−/−^ mice [127]. Indeed, miR-126 released by endothelial cells controls VSMC behavior [126] and limits neointimal formation [129]. Given that circulating miR-126 expression is altered in several cardiovascular diseases [130], these findings suggest that estradiol's vasculoprotective and antiatherogenic properties could be partly mediated by miR-126. Circulating miR-126 is downregulated in different cardiovascular diseases [131, 132], although its relationship with and ageing is contradictory; compared to young individuals, miR-126 was downregulated in blood samples from centenarians [133], but miR-126-5p expression in circulating exosomes was higher in postmenopausal versus premenopausal women [122] and miR-126-3p was increased in senescent endothelial cells in vitro and in plasma collected from healthy older patients [134]. Olivieri et al. hypothesize that this miR-126 upregulation is a compensatory mechanism to reduce cell dysfunction during normal ageing.

*miR-106* expression is sex-specific. miR-106a/b were among the sex-specific miRNAs regulated via ER*β* in a murine model of pressure overload-induced cardiac fibrosis mediated by regulation of specific profibrotic MAPK signaling repressors [135], evidence that miR-106 may be involved in sex-related differences in this pathology. In estradiol-treated rat cardiac fibroblasts, miR-106b expression is decreased in both female and male cells while miR-106a expression is downregulated in female cells but upregulated in male cells [135]; furthermore, miR-106b was downregulated in cultured VSMCs treated with estradiol [97], suggesting that estradiol has sex-specific effects in downregulating miR-106b. In contrast, miR-106b-5p expression was lower in postmenopausal women not using HRT compared to those that did use HRT [122], suggesting that estrogens have the opposite effect on miR-106 expression during ageing. The downregulation of miR-106a has been also reported in replicative endothelial cell ageing [136] and in serum from older women [137]. In endothelial cells treated with tumor necrosis factor alpha (TNF-*α*), miR-106b-5p exerts antiapoptotic effects by repressing phosphatase and tensin homolog *(PTEN)* caspase activity [138], and in a rat cardiomyoblast cell line exposed to hypoxia, it suppresses apoptosis by directly targeting p21 [139], findings that concur with the known repressive effects of estrogens on *PTEN* and apoptosis [140, 141].

In *miR-221/222*, the cluster containing the miR-221/222 gene is located on the X chromosome and is regulated both by ageing and estrogen-mediated mechanisms; serum levels of miR-222 increase with ageing in both males and females [137]. Moreover, in human aortic endothelial cells, miR-221/222 expression was upregulated in an in vitro replicative senescence model and correlated with decreased *eNOS* expression and activity [142]. Moreover, endothelial cells transfected with miR-221 and miR-222 showed reduced eNOS protein levels [106]. However, eNOS 3′-UTR mRNA does not contain a target sequence for miR-221 or miR-222, suggesting that an intermediate mechanism may be responsible for this miR-221/222-mediated *eNOS* repression. Indeed, miR-222, that was among the sex-dimorphic miRNAs identified in mice heart tissues and isolated cardiomyocytes, inhibits *eNOS* expression by directly targeting ets-1 mRNA [143], an upstream *eNOS* regulator. These results indicate that miR-222 plays an important role in heart function that may be sex-specific in terms of providing female cardioprotection [144]. Conversely, miR-221/222 regulate and are regulated by ER*α* activity [145], suggesting the presence of a negative regulatory loop between them. One hypothesis is that before menopause, estrogen-ER*α* activity limits miR-221/222 levels, thus maintaining *eNOS* expression and cardioprotection. However, ageing and menopause increase miR-221/222 levels and decrease ER*α* and eNOS expression by downregulating ets-1. In addition, miR-221/222 are strongly upregulated in the carotid artery in a balloon injury model and their depletion suppresses VSMC proliferation and neointimal formation [146] which may explain the role of estrogens in attenuating vascular damage [86].

*miR-143/145* activity appears to be essential in regulating VSMCs to prevent the ageing-related switch from a contractile to synthetic-proliferative VSMC phenotype [147], and miR-143/145 dysregulation has been described in different cardiovascular pathologies. These miRNAs maintain VSMCs in a quiescent state and inhibit proliferation by regulating several targets, including angiotensin-converting enzyme [148] which modulates vascular contractibility. It has been reported that miR-143/145 are delivered from endothelial cells to VSMCs where they repress genes associated with dedifferentiation [149] such as myocardin, *ELK1*, and *KLF4*. Estradiol induces miR-143/145 expression in pulmonary artery smooth muscle cells (PASMCs) via specific ER binding sites located in their promoter regions [150]. Moreover, estradiol-treated PASMCs secrete exosomes enriched in miR-143/145 which regulate VSMC-endothelium crosstalk in pulmonary arterial hypertension, regulating vascular remodeling [150] and perhaps playing a role in exerting estrogen-mediated effects on the VSMC phenotype. Although no specific studies on miR-143/145 expression in cardiovascular cells during ageing have been undertaken, miR-145 expression negatively correlates with age in patients with coronary artery disease [151].

*miR-30* is estrogen-sensitive and its expression is related to estrogen levels in different tissues [110, 152, 153]; indeed, ER binding sites have been identified upstream of the miR-30 transcription start site [96, 98]. Reinforcing the role of estrogens in regulating miR-30b-5p, its expression was downregulated in serum from ovariectomized rats and from postmenopausal osteoporotic women [115] and miR-30b-5p was the most upregulated miRNA in endothelial cells treated with estradiol [98]. There is also evidence that miR-30b family members are involved in cardiovascular diseases because their expression is decreased in vascular injury [154], inversely correlates with blood pressure parameters [155], and inhibits VSMC proliferation and neointimal formation [154]. However, the role of miR-30 family members in myocardial infarction remains controversial [156–158]. In endothelial cells, miR-30 is implicated in angiogenic processes [159], has anti-inflammatory effects by decreasing angiopoietin 2-induced *VCAM1* expression [160], and inhibits apoptosis in human coronary artery endothelial cells [161]. In this regard, it is noteworthy that estradiol also has proangiogenic, anti-inflammatory, and antiapoptotic properties in response to vascular injury [162].

*miR-23a* belongs to the miR-23-24-27 cluster which has been implicated in angiogenic processes and cardiac function [163] and also has ER*α* binding sites in its regulatory regions. Members of this cluster are upregulated during endothelial replicative ageing [164], and miR-23a overexpression reduces telomere length and induces senescence in human fibroblasts [165]. In serum and myocardium in rats, miR-23a expression inversely correlates with estradiol levels and regulated connexin-43 in a menopausal rat model [166]. In postmenopausal rats, estrogen supplementation rescued blockage of cardiac conduction by decreasing miR-23a, thus revealing potential mechanisms involved in postmenopause-related arrhythmias [167]. Moreover, miR-23a may affect cardiac hypertrophic processes [168]: its increased expression in estrogen-deficient ovariectomized mice causes mitochondrial compromise and ventricular remodeling by directly repressing peroxisome proliferator-activated receptor-*γ* coactivator 1-*α (PGC*-*1α)* expression in cardiomyocytes [169]. Moreover, miR-27 is upregulated in senescent endothelial cells [164] and in circulating exosomes in premenopausal versus postmenopausal women [122]. This miRNA has been implicated in LDLR expression [170], angiogenic processes [107], and may be a biomarker for progression in asymptomatic carotid stenosis [171].

*miR-203* is upregulated in estradiol-treated mouse aortic VSMCs [97]; miR-203 induction is regulated through an ER*α*-dependent mechanism and its inhibition abolishes estradiol-mediated inhibition of VSMC proliferation, suggesting a role for miR-203 in the antiproliferative effect of estrogens [97]. In addition, miR-203 can downregulate ER*α* by direct targeting [172], suggesting its involvement in a regulatory loop. Conversely, increased miR-203 expression found in aged aorta has recently been associated with age-related VSMC stiffness [173].

In *miR-144*, circulating estradiol concentrations were positively correlated with miR-144-5p in a study of women receiving HRT [122], and in the same study, miR-144-5p was inversely correlated with TNF-*α* levels, together suggesting it has an estrogen-mediated role in regulating inflammatory processes. Indeed, miR-144 is implicated in targeting superoxide-related proteins such as COX-2 [174] and the NADPH oxidase component Rac1 [175]. Conversely, miR-144 is upregulated in cerebral microvascular endothelial cells from aged mice, and its inhibition upregulates the antioxidant transcription factor NRF2 [176]; miR-144 is also involved in lipid metabolism by repressing the expression of cholesterol efflux regulatory protein ABCA1 [177], while the ABCA1 inducer, nuclear factor LXR, increases miR-144 expression, suggesting the presence of a negative regulatory loop. Furthermore, induced LXR and ABCA1 expression has been related to an estradiol-dependent reduction of lipid accumulation in macrophages and VSMCs [58, 59] suggesting that estrogen tightly regulates lipid content via this miRNA-mediated pathway. Finally, the effect of estrogen on cholesterol efflux is modified by age [178] and this may be related with the dysregulation of miR-144 expression during ageing.

*miR-146a* is one of the key miRNAs associated with vascular inflammation; it regulates endothelial activation by targeting upstream NF-*κ*B pathway regulators such as TNF receptor associated factor *(TRAF)* 6 and IL-1 receptor associated kinase *(IRAK1)* [179] and is described as a marker of senescence-associated proinflammatory status in endothelial cells [180]. In a replicative endothelial cell model, miR-146a downregulation may have caused endothelial senescence by increasing the expression of its direct target, *NOX4* [181]. The relationship between miR-146a expression and estrogen has previously reported since circulating miR-146a expression was different between HRT-treated and nontreated postmenopausal genetically identical twins [121]. In addition, estradiol inhibition of miR-146a expression may be a key regulator of lipopolysaccharide-induced interferon-gamma *(IFN*-*γ)* expression in lymphocytes [128].

*miR-21* expression is also related to estrogens and ageing; increased miR-21 levels have been found in aged heart tissues [182], in senescent vascular cells [164], and in blood samples in elderly people [183]. However, conversely, repression of miR-21 in senescent human aortic endothelial cells seems to increase the expression of antiproliferative and proapoptotic molecules [142] and may represent a diagnostic biomarker for acute myocardial infarction [184]. In cardiac fibroblasts, miR-21 action is implicated in heart hypertrophy by activating MAPK pathways [185], proinflammatory responses in endothelium under oscillatory shear stress [186], and VSMC proliferation after vascular injury [187]. However, the cardiovascular effects of estrogens attenuate these responses; indeed, estradiol downregulates miR-21 by binding to ERs in its promoter region [188] and miR-21 expression in postmenopausal women using HRT was lower than their twin sisters who did not use HRT [121]. Finally, in a murine model of pressure overload-induced cardiac fibrosis, miR-21 was among sex-specific miRNAs regulated by ER*β* and was downregulated in estradiol-exposed primary cardiac fibroblasts [135] which may explain sex-dependent differences in cardiac remodeling [189].

*miR-34* is part of a well-described family of ageing-associated miRNAs related to cardiomyocyte apoptosis [190] and vascular cell senescence [191, 192]. The expression of miR-34 is elevated in old versus young aorta [173] as well as during ageing and in cardiac pathologies; however, inhibition of the miR-34 family improves heart function [193]. Moreover, miR-34 is influenced by sex and estrogens; miR-34b-3p expression is higher in male rather than female mouse hearts [143]. Furthermore, estradiol exposure decreases miR-34a expression in cultured endothelial cells [194] and is associated with SIRT1-dependent regulation of *eNOS*. Moreover, blockage of miR-34a expression was more effective in females than in males in a murine model of moderate dilated cardiomyopathy [195], highlighting the importance of studying sex-related differences in miRNA-based therapies.

*miR-22* is upregulated in aged hearts [196, 197] and contributes to cardiac hypertrophy by promoting senescence and migratory activity in cardiac fibroblasts [196] and by inhibiting autophagy in cardiomyocytes [197]. Estradiol also appears to decrease miR-22 expression in primary cardiomyocytes via ER*α*-mediated mechanisms [198]. In addition, miR-22 downregulation increases oxidative defense by increasing expression of its direct target, Sp1 [198]: a regulatory pathway that may explain estradiol-mediated cardioprotection. A reciprocal feedback loop between ER*α* and miR-22 may be involved in fine-tuning the regulation of estrogen action by posttranscriptionally controlling ER*α* expression [199].

*miR-125* is part of a family of miRNAs which are mainly associated with the activation of inflammatory cells. In macrophages exposed to lipopolysaccharide (LPS), miR-125a is upregulated [200] and miR-125b is downregulated [201]. However, more studies are needed to understand their role in activating or inhibiting inflammatory processes. The regulation of some miR-125 family members is estrogen-dependent: estradiol inhibits NF-*κ*B activity by restoring downregulated miR-125b expression in LPS-stimulated macrophages [201] and both miR-125a and miR-125b are downregulated in lymphocytes exposed to estradiol [128]. Moreover, miR-125 activity is also associated with angiogenesis during ageing: miR-125a-5p expression is increased in endothelial cells from aged mice and is involved in impaired angiogenesis [202].

## 6. Conclusion

**Figure 1:**
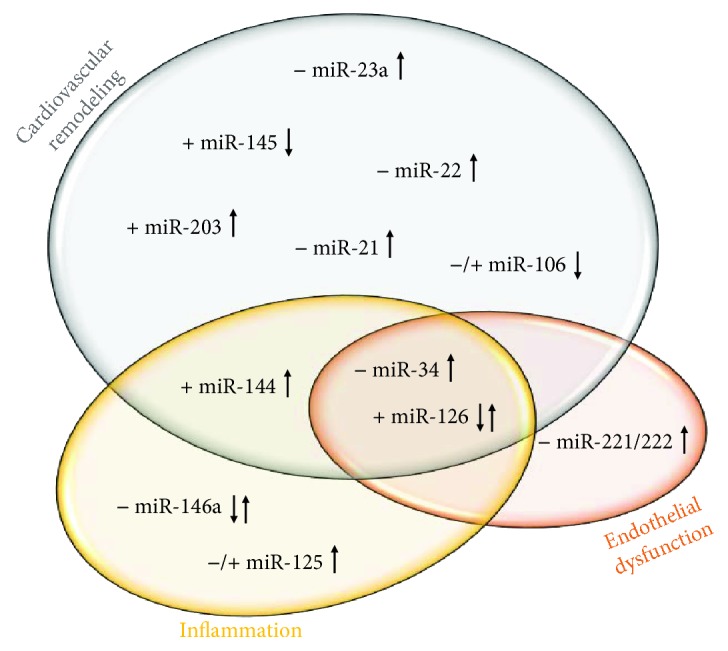
Estrogen-sensitive miRNAs associated with cardiovascular ageing are classified according to their role in the three main mechanisms leading to cardiovascular ageing: cardiovascular remodeling, endothelial dysfunction, and inflammation. (↓) and (↑) indicates increased or decreased expression in cardiovascular tissues during ageing, respectively. Role of estrogens on the expression of represented miRNA is also represented; (−) and (+) indicates negative or positive regulation, respectively. Controversial findings dependent on expression in different tissues/cells are indicated as (↓↑) and (−/+).

The role of sex hormones in cardiovascular physiology has been extensively studied and has been proposed as the cause of the reported sex-related differences in cardiovascular diseases. Vasodilation, inflammation inhibition, and the action of antioxidants have all been attributed to the effect that estrogens exert on the cardiovascular system. However, the effects that estrogens have on the heart and vascular tissues are themselves modified by ageing. Estrogens modify cardiovascular function by modulating gene expression, and in this sense, miRNAs have emerged as a new regulatory mechanism of both physiological and pathological processes because they regulate gene expression profiles at the posttranscriptional level (Figure 1). Studies on miRNAs have provided insights into cardiovascular function, age-associated physiological changes, and cardiovascular pathologies. However, information about the role of miRNAs in estrogen-dependent processes in cardiovascular ageing is still scarce. Therefore, basic research to analyze sex-specific miRNA regulation can help us to understand differences in cardiovascular diseases between men and women. It may be possible to translate this new knowledge into clinical research, using miRNAs as potential tools for giving diagnoses and/or prognoses, as affordable and noninvasive biomarkers, and as a therapeutic tool for regulating (silencing or increasing) miRNA levels. Thus, future perspectives in miRNA-based therapies should consider the importance of sex-related differences in vascular ageing.

## Figures and Tables

**Table 1:** HRT-sensitive miRNAs involved in age-associated diseases in postmenopausal women; miRNAs related to disease and the tissue sample types used in the study are listed and the miRNA-related function and specific miRNA targets are shown.

Disease (tissue)	miRNA	Function (targets)	References
Osteoporosis (bone)	miR-127 and miR-136	Regulation of bone mass (COL1, ALP, RUNX2, and OC)	[114]
Osteoporosis (serum)	miR-30b-5p	Biomarker	[115]
Sarcopenia (skeletal muscle)	miR-182 and miR-223	Regulation of the insulin/IGF-1 pathway (IGF-1R and FOXO3A)	[116]
Obesity (adipose tissue)	miR-19a-3p	Adipocyte cell fate, death, and proliferation (ESR1, AKT1, BCL2, BRAF, and CCND1)	[117]
Inflammageing (Serum)	miR-21 and miR-146a	Biomarker	[121]

**Table 2:** Circulating miRNAs associated with estrogen levels and/or ageing.

miRNA	Ageing	Estrogen	Sample/model	References
miR-126	−		Blood samples from centenarians	[133]
miR-126-3p	−		Plasma from healthy people	[134]
miR-126-3p		+	Serum from healthy women	[127]
miR-126-5p	+		Serum exosomes from postmenopausal women	[122]
miR-106b	−		Serum from older women	[137]
miR-106b-5p		+	Serum from HRT-treated postmenopausal women	[122]
miR-30b-5p		+	Serum samples from ovariectomized rats	[115]
miR-23a		−	Serum from female rats	[166]
miR-144		+	Serum from HRT-treated postmenopausal women	[122]
miR-146a		−	Serum from HRT-treated postmenopausal women	[121]
miR-21	+		Plasma from elderly people	[183]
	+	−	Serum from HRT-treated and non-HRT-treated postmenopausal women	[121]

Positive or negative associations with ageing and estrogens are represented, and the sample type or model used in each study is shown. HRT: hormone replacement therapy.

**Table 3:** Cardiovascular-related miRNAs associated with estrogens and ageing.

miRNA	Ageing	Estrogen	Tissue/cell	Function (target)	References
miR-126		+	Endothelial cells	Endothelial proliferation, migration, tube formation, and monocyte adhesion (Spred1 and VCAM1)	[127]
	+		Endothelial cells	Senescence-associated compensatory mechanism (Spred1)	[134]
		−	Lymphocytes	Unexplored	[128]

miR-106	−		Endothelial cells	Unexplored (p21/CDKN1A)	[136]
		−	VSMCs	Unexplored	[97]
		−	Cardiac fibroblasts	Regulation of cardiac fibrosis via ER*β* (Rasa1 and Rasa2)	[135]

miR-221/222	+		Endothelial cells	Suppression of eNOS and p^Ser1177^-eNOS	[142]
	+		Aorta	Unexplored	[173]
		f < m	Cardiomyocytes	Regulation of eNOS expression (Ets-1)	[143]

miR-143/145		+	PASMCs	SMC and EC cell migratory phenotypes	[150]

miR-30b-5p		+	Endothelial cells	Unexplored	[98]

miR-23a	+		Endothelial cells	Unexplored	[164]
	+		Fibroblasts	Telomere dysfunction (TRF2)	[165]
		−	Myocardium	Loss of cardiac gap junctions (CX43)	[166]
		−	Cardiomyocytes	Ventricular remodeling (PGC1)	[169]

miR-203	+		Aortic SMCs	VSMC stiffness (Src and ERK)	[173]
		+	Aortic SMCs	Inhibition of VSMC proliferation (Abl1 and p63)	[97]

miR-144	+		Endothelial cells	Antioxidant response (NRF2)	[176]

miR-146a	+		Endothelial cells	Proinflammatory status marker (IRAK1)	[180]
	−		Endothelial cells	Senescence-like phenotype (NOX4)	[181]
		−	Lymphocytes	Regulation of LPS-induced IFN-*γ* (unknown)	[128]

miR-21	+		Heart	Unexplored	[182]
	+		Endothelial cells	Decrease angiogenesis and cell proliferation (NFIB and CDC25A)	[164]
	−		Endothelial cells	Antiproliferative effect (associated with PTEN and p27)	[142]
		−	Cardiac fibroblasts	Regulation of cardiac fibrosis (SPRY1, Rasa1, and Rasa2)	[135]

miR-34	+		Aorta	Unexplored	[173]
	+		Aorta/VSMCs	Promotes VSMC senescence and inflammation (SIRT1)	[192]
	+		Endothelial cells	Cell growth arrest and senescence (SIRT1)	[191]
	+		Cardiomyocytes	Age-associated cell death (PNUTS/PPP1R10)	[190]
		−	Endothelial cells	Regulation of eNOS expression (SIRT1)	[194]

miR-22	+		Cardiac fibroblasts	Induction of cellular senescence and migratory activity (OGN)	[196]
	+		Cardiomyocytes	Inhibition of cardiac autophagy and cell hypertrophy (Akt3, Hdac6, and Ppara)	[197]
		−	Cardiomyocytes/myocardium	Increased antioxidant defense (SP1)	[198]

miR-125	+		Endothelial cells	Impaired angiogenesis (RTEF1)	[202]
		+	Macrophages	Inhibitor of NF-*κ*B signaling (*κ*B-Ras2)	[201]
		−	Lymphocytes	Unexplored	[128]

Positive or negative associations with ageing and estrogens are represented, and the tissue or cell type used, miRNA-related function, and described miRNA target are shown. Sex differences are indicated where appropriate (f: female; m: male). SMC: smooth muscle cell; VSMC: vascular SMC; PASMC: pulmonary artery SMC.
